# First case of HIV cure in a woman after stem cell transplantation reported at CROI-2022

**Published:** 2022-04

**23 March 2022** - The International Maternal Pediatric Adolescent AIDS Clinical Trial Network (IMPAACT) P1107 reported the first case of HIV cure in a woman living with HIV submitted to a dual stem cell transplant (i.e., an umbilical cord blood transplant combined with a half-matched bone marrow transplant) for treatment of an acute myelogenous leukemia. The IMPAACT P1107 researchers presented the case details during the oral abstract session held at the 29th Conference on Retroviruses and Opportunistic Infections (CROI 2022). The study participant is a woman from New York (USA) who stopped antiretroviral therapy (ART) at 37 months post-transplant and has had no HIV detected for 14 months. The dual stem cell therapy also led to remission from leukemia that she developed in 2017.

The IMPAACT P1107 is an observational study that aims to describe the outcomes in people living with HIV who undergo a transplantation with cord blood stem cells with a CCR5 genetic mutation for treatment of cancer, hematopoietic disease, or other underlying disease. This genetic mutation results in T cells without CCR5 co-receptors. As HIV needs to use these co-receptors to infect T cells, the rationale of the study is that chemotherapy given to people with cancers or other illness, followed by a transplant using stem cells that carry this CCR5 mutation, can change the immune system to make it genetically resistant to HIV.

HIV remission, or cure, resulting from stem cell transplants had been previously reported in 2 cases. The first case, known as the Berlin patient (a man with acute myelogenous leukemia), was reported in 2009. He was submitted to a bone marrow stem cell transplant and experienced HIV remission for 12 years. He died of recurrent leukemia in September 2020. The second case, known as the London patient (a man with Hodgkin lymphoma), was reported more recently and has been in HIV remission for more than 30 months after a bone marrow stem cell transplant. This third case of HIV remission, now documented in a woman, suggests that a dual stem cell transplantation strategy could also be considered as an option to achieve HIV remission and cure for people living with HIV who require a stem cell transplant for other diseases.

This case further supports the proof of concept for an HIV cure using stem cell transplants, but it is important to highlight that this approach is an invasive, complex and risky medical procedure and still not viewed as a feasible strategy to scale up to the millions of people living with HIV globally. Furthermore, as this CCR5 mutation is very rare (around 1% of general population), the chances of finding a suitable stem cell donor are very low.

“Despite of feasibility challenges, this new HIV remission case is very exciting news and will continue to energize the HIV cure research agenda, reminding us of its potential to beat HIV,” says Dr Meg Doherty, Director of WHO’s Global HIV, Hepatitis and STI Programmes.

More details about the case

The HIV cure case described at the CROI 2022 involves a middle-aged woman of mixed-race ancestry who had developed high-risk acute myeloid leukemia while on ART, 4 years after a diagnosis of acute HIV infection. She achieved the leukemia remission after conventional leukemia chemotherapy and her HIV disease was also well controlled but with detectable virus. In 2017, she received a transplant of umbilical cord blood stem cells with a CCR5 mutation, supplemented with donor’s stem cells from the bone marrow of an adult relative. After receiving this dual stem cell transplant, she was engrafted with 100% cord blood cells at day 100 and had no detectable HIV. At 37 months post-transplant, the patient stopped ART. According to study team, no HIV was detected in the patient for 14 months except for a short transient detection of trace levels of HIV DNA in the patient’s blood cells at 14 weeks after stopping ART.

About the dual stem cell transplant approach

The dual stem cell (or haplo-cord) transplant is a new transplant strategy that involves the transfusion of stem cells from an umbilical cord of neonates, complemented with stem cells from the bone marrow of an adult donor. This dual transplant process has been used in some individuals with high-risk cancers and requires less restrictive human leucocyte antigen (HLA) sample matching than adult- stem cells-only transplants, and it also makes the transplant procedure faster and safer. In this case, the cells of the umbilical cord had the CCR5 mutation and the adult stem cells also didn’t require an identical HLA matching, which is especially difficult to get for patients of African or mixed-race ancestry.

Available from: https://www.who.int/news/item/24-03-2022-first-case-of-hiv-cure-in-a-woman-after-stem-cell-transplantation-reported-at-croi-2022

